# Longitudinal Evaluation of Vestibular Symptoms in Patients with Vestibular Schwannoma After Robotic-Guided Stereotactic Radiosurgery Using the Dizziness Handicap Inventory (DHI)

**DOI:** 10.3390/jcm14020299

**Published:** 2025-01-07

**Authors:** Daniel Rueß, Susanne Vojacek, Eda Güngör, Jan Christoffer Lüers, Stefan Hunsche, Karolina Jablonska, Martin Kocher, Maximilian I. Ruge

**Affiliations:** 1 Department of Stereotactic and Functional Neurosurgery, Centre for Neurosurgery, Medical Faculty, University of Cologne, 50933 Cologne, Germany; susanne.vojacek@gmx.de (S.V.); eda.guengoer@uk-koeln.de (E.G.); stefan.hunsche@uk-koeln.de (S.H.); martin.kocher@uk-koeln.de (M.K.);; 2 Department of Otorhinolaryngology, Head and Neck Surgery, Medical Faculty, University of Cologne, 50933 Cologne, Germany; jan-christoffer.lueers@uk-koeln.de; 3 Department of Radiation Oncology, Cyberknife Centre, Medical Faculty, University of Cologne, 50933 Cologne, Germany; karolina.jablonska@uk-koeln.de

**Keywords:** radiosurgery, vestibular schwannoma, vertigo, dizziness, DHI, Cyberknife

## Abstract

**Background**: Vestibular symptoms can severely affect patients with vestibular schwannomas (VSs). Studies assessing vestibular symptoms beyond clinical routine assessment in patients with VS treated by stereotactic radiosurgery (SRS) are scarce. Therefore, we employed the standardized questionnaire Dizziness Handicap Inventory (DHI) to systematically evaluate vestibular symptoms prior to and after SRS. **Methods**: For this retrospective single center study, we included patients who received Cyberknife^®^ SRS for newly diagnosed unilateral VS between 2012 and 2022, and who had a minimum of two follow-up (FU) visits. Besides clinical assessment, the presence and severeness of vestibular symptoms before and after treatment was recorded by using the DHI. Overall DHI symptom scores (1–100) were classified into four grades (0 = “none”, 1 = “mild”, 2 = “moderate” and 3 = “severe”). The results were correlated with tumor-, patient-, and treatment-related characteristics. **Results**: We analyzed 128 patients with a median age of 60 years (range: 20–82) and a median FU of 36 months (range: 11–106 months). The median tumor volume was 0.99 cm^3^ (range: 0.04–7.1 cm^3^). A median marginal dose of 13 Gy (range: 12–14 Gy) was administered. The crude rate of local tumor control was 99.2%. The mean DHI total score at last follow-up (LFU, 25.5 ± 24.7; range 0–92) was significantly lower than before SRS (29.4 ± 25.3; range:0–92, *p* = 0.026), which was reflected in a higher proportion of patients with DHI grade “none” and a lower proportion of patients with DHI grade “severe” at LFU. Chi-square tests showed a significant correlation of the DHI grades (DHI 0–1 vs. DHI 2–3) with the absence or presence of vestibular symptoms both before SRS (*p* < 0.001, CI 95%) and at LFU (*p* = 0.038). **Conclusions**: The DHI is a feasible and valid instrument for measuring vestibular symptoms after SRS. In addition, the DHI enables the quantification of symptoms and can therefore serve as an important tool for outcome assessment after SRS of VS. In the present cohort, DHI scores improved significantly during FU.

## 1. Introduction

Vestibular schwannomas (VSs) represent 8% of all intracranial tumors and, with an incidence of 19.4 per million, and are classified as benign central nervous system (CNS) tumors [1]. Stereotactic radiosurgery (SRS) has become an established procedure for the treatment of VS since the first publication by Hirsch et al. in 1979 [2]. This therapeutic option has emerged as the first-line treatment, particularly for small symptomatic and/or growing tumors, as also recommended in 2020’s EANO guidelines proposed by the “vestibular schwannoma task force” of the EANO (European Association of Neuro-Oncology) [3].

VSs are usually clinically characterized by unilateral hearing loss (94%) and unilateral tinnitus (68%). The incidence of vestibular symptoms such as dizziness and balance disorders varies widely (17–75%) and is clinically underreported [4,5]. In VS patients, local tumor control and hearing preservation are generally reported as the main outcomes, as these parameters can be objectified reliably and reproducibly using valid measurement methods.

However, previous studies have shown that continuous dizziness is a strong predictor of disability and reduced quality of life in patients with VS [6,7,8,9]. Hence an objective clinical evaluation of balance disorders and vertigo symptoms in VS patients often is challenging due to the non-specific symptoms that are described by the patient. Therefore, available studies investigating outcome after radiosurgery (SRS) with valid objective data addressing vestibular symptoms beyond subjective patient reports are scarce [10], especially after robotic-guided SRS. In order to systematically evaluate the incidence and severity of vestibular symptoms, we introduced a well-studied PROM (Patient-Reported Outcome Measure) to assess and quantify vestibular symptoms after SRS. The 25-item Dizziness Handicap Inventory (DHI) is the most commonly used questionnaire for clinical assessment of impairment caused by balance disorders and vertigo symptoms [11,12].

The aim of this study was to (i) investigate whether the DHI can be proved to be a feasible and valid tool for measuring vestibular symptoms before and during follow-up after robotic SRS. Furthermore, (ii) we intended to elucidate patient’s individual longitudinal course of potential vestibular symptoms prior to, at 6-month follow-up, and at last available follow-up after robotic-guided SRS, and (iii) to correlate these findings with clinical and treatment-related parameters.

## 2. Material and Methods

### 2.1. Patient Selection

All patients who (i) were treated in our institution between November 2012 and April 2022 for a unilateral VS with robotic-guided SRS by Cyberknife, (ii) who had at least two clinical and radiological follow-up visits ≥ 6 months after SRS treatment, and (iii) who completed the DHI questionnaire correctly before SRS, at the 6-month FU and at their individual last FU were included for this retrospective analysis.

### 2.2. Data Acquisition

The present retrospective analysis at a single center was conducted in accordance with the institutional rules of the local ethics committee. All clinical data were retrieved from patient’s (electronic) records. We documented age, gender, date of SRS treatment, tumor volume, Koos grade, type of pre-treatment, and date of follow-up exams including MRI and clinical findings (initial and during follow-up). The presence or absence of vestibular symptoms was assessed using subjective criteria (e.g., patient-reported dizziness) and clinical examinations before and after SRS. In addition, relevant irradiation parameters such as irradiation doses (surface, minimum, maximum, and mean dose in Gy, isodose, coverage, and nCI) were determined.

### 2.3. Evaluation of Vestibular Symptoms Using the DHI

To assess patient’s vestibular symptoms, we applied the DHI questionnaire, which is now routinely used as part of our standardized evaluation battery for patients with VS prior to and during follow-up after SRS. Each question belongs to one of 3 subgroups which represent functional, emotional, and physical aspects of vertigo and balance disorders [12]. The possible responses to each item are either “no”, “sometimes”, or “yes”. A “no” is scored 0 points, a “sometimes” is scored 2 points, and a “yes” is scored 4 points.

Since there are a total of 25 questions, the maximum score is 100 points, indicating the maximum self-perceived dizziness handicap. The minimum score is 0, meaning that no handicap due to dizziness or balance disorders is perceived. According to Jacobsen et al. [12], a minimum change of 18 points is considered as a true change in DHI score. Furthermore, the DHI score results in four severity grades from 0 to 3, a score of 0 to 14 indicating “none”, 16 to 34 “mild”, 36 to 52 “moderate”, and 54 or greater designating “severe” handicap.

### 2.4. SRS Treatment Planning and Delivery

The VS and potentially vulnerable adjacent structures (e.g., brainstem, cerebellum, trigeminal nerve) were contoured on a planning CT (Toshiba, Tokyo, Japan, Aquilon 16-slice multidetector CT) with a 1 mm slice thickness and on a defined set of MRI series comprising contrast-enhanced T1, T2, and FLAIR weighted images (Phillips, Amsterdam, The Netherlands, MR-Scanner, 1.5 or 3 Tesla) registered to the planning CT. The contouring was performed by neurosurgeons experienced in radiosurgery. For robotic Cyberknife SRS, the patient was immobilized on the robotic Cyberknife treatment table (Accuray, Sunnyvale, CA, USA) by means of a custom-made aquaplast mask. For treatment planning, the software Multiplan v4.5 (since 2016 v4.6) was used. The final irradiation plan was evaluated in an interdisciplinary consensus meeting between the stereotactic neurosurgeon, a radiation oncologist experienced in SRS, and a medical physicist.

### 2.5. Statistical Analysis

For descriptive statistics, continuous values are given as the median and range or mean and standard deviation; ordinal and categorical variables are shown as counts and percentage. Descriptive summaries were prepared for the patients’ demographics and radiation parameters. A paired *t*-test (Wilcoxon matched-pairs signed rank test) was used to compare the DHI total score before and after treatment. An unpaired *t*-test (Mann–Whitney test) was used to compare subgroups of patients with or without vestibular symptoms. For correlation analysis between vestibular symptoms assessed during clinical follow-up and the four DHI grades (none, mild, moderate, and severe) before and at LFU, a chi-square test was used. Furthermore, correlation analysis with regard to the true change in DHI score was conducted using a binary logistic regression. A *p*-value of <0.05 was considered statistically significant. The statistical analysis was performed using the software Graphpad PRZM 10 and SPSS 29.0.1 (IBM Statistics, Chicago, IL, USA).

## 3. Results

### 3.1. Patient Cohort and Tumor Characteristics

A total of 128 patients (female/male = 54/74) with a median age of 60 years (range 20–82 years) were analyzed (Table 1). Overall median follow-up was 36 months (range, 12–106 months) and mean follow-up was 42 ± 25.9 months. The median marginal dose delivered to all tumors was 13 Gy (range, 12–14 Gy). The median prescription isodose was 80 (range, 65–82%). According to the Koos classification, about two-thirds (n = 79, 62%) of the patients had Koos grade I or II (Table 1). The overall average tumor volume was 1.33 ± 1.3 cm^3^, and the median was 0.95 cm^3^ (range 0.04–7.1). Based on previously suggested criteria according to Rueß et al. [13], one patient (0.8%) experienced tumor progression 44 months after SRS. He was retreated with a second radiosurgery (prescribed dose: 13 Gy to an 80% isodose in one fraction). The other patients in the collective showed stable tumor size or regression. The most frequent symptom prior to SRS was hearing impairment (n = 101, 79%). Overall, 11% of the patients (n = 14) had a complete loss of hearing. Seventeen patients (13%) were treated for recurrent VS following previous surgery. Of these patients, eleven had anacousis and twelve had facial nerve palsy, which remained stable during FU after SRS.

### 3.2. DHI Scores and Vestibular Function

Eighty-one (63%) of the patients had vestibular symptoms prior to SRS, which clinically improved in 25% (n = 20) after SRS. In contrast, 21 patients (26%) reported a deterioration in vestibular symptoms after SRS. About one-third of the patients (n = 47, 36%) did not show vestibular symptoms before SRS. Twelve (25%) of these patients experienced transient (n = 9) or permanent (n = 3) new onset of vestibular symptoms.

**Figure 1:**
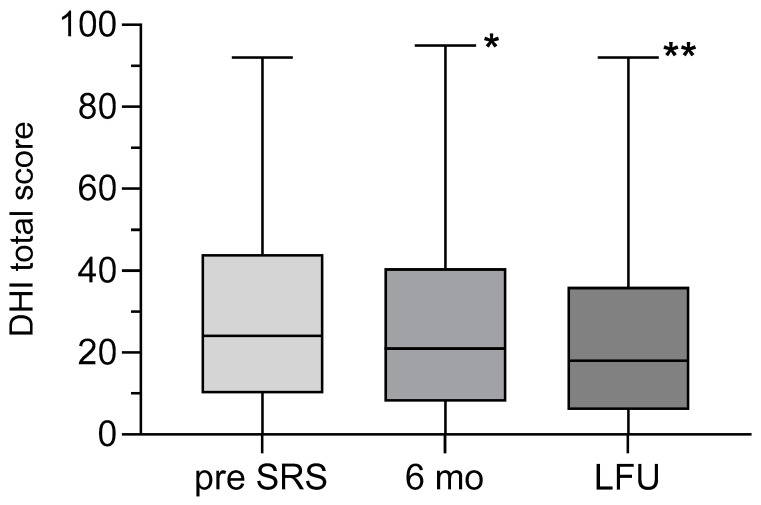
The figure shows a box–whisker plot with the median DHI total score before SRS (pre SRS), after six months (6 mo), and at LFU. The mean DHI total score before SRS was significantly reduced at six months and at LFU (* Wilcoxon matched-pairs signed rank test, * *p* = 0.038 and ** *p*-value = 0.0058).

All patients completed the DHI as required at the defined time points, demonstrating the feasibility of this measure. The mean DHI total score was 29.4 ± 25.3 (range: 0–92) before SRS and 25.5 ± 24.7 (range: 0–92) at LFU. The decrease in DHI total score was statistically significant (*p* = 0.0058) (Figure 1). Before SRS, out of 73 patients with a DHI score above 18 points, an improvement (minimum decrease of 18 points) was seen in 11% (n = 8/73) after 6 months and in 30% (n = 22/73) at LFU. In contrast, 120 patients had a DHI under 82 points and a true worsening of DHI (minimum increase of 18 points) was seen in 2.5% (n = 3/120) after 6 months and 9% (n = 11/120) at LFU. The binary logistic regression model did not show any influences of several variables (Table 2) on the (log) odds of DHI improvement or deterioration at LFU. In the 17 pre-operated patients, only two patients showed a significant improvement in DHI in FU, while the remaining 15 pre-operated patients showed a largely stable DHI value after SRS.

**Figure 2:**
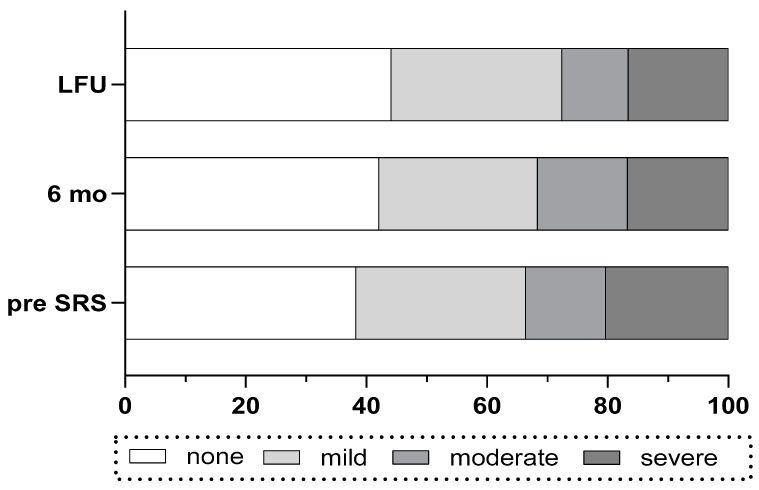
Comparison of the four categories of DHI before (pre SRS), at six months and at LFU.

**Figure 3:**
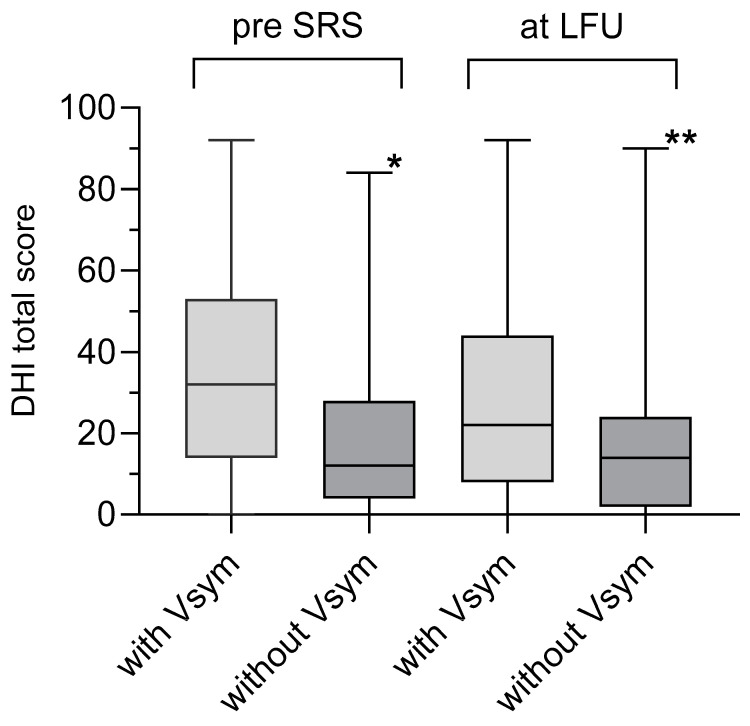
The figure shows a box–whisker plot with the median DHI total score of patients with or without Vsym and before SRS (pre SRS) and at LFU. As expected, before SRS, the mean DHI total score of patients with Vsym was significantly higher (Mann–Whitney test, * *p* = 0.0004) than of patients without Vsym. The same was true for the patients at LFU (Mann–Whitney test, ** *p* = 0.0032).

Before SRS, most patients had a DHI grade of “none” (n = 48) or “mild” (n = 36), and a lower number of patients had “moderate” (n = 17) or “severe” (n = 26) (Table 1). During FU, in 33% of the patients (n = 27 of 80), the DHI improved by one or more grades, whereas in 17% (n = 17 of 102) of the patients, deterioration by one or more of DHI grade was observed. Overall, the number of patients with a “none” grade of DHI increased and the number of patients with a “severe” DHI score decreased during the FU period (Figure 2). The incidence of vestibular symptoms before and at LFU correlated well with the grades of DHI (chi-square *p* = 0.002, Table 3). Likewise, the group of patients with vestibular symptoms before SRS had a significantly higher mean DHI total score (34.9 ± 2.9) than the group of patients without vestibular symptoms (20 ± 3.1, unpaired *t*-test, *p* < 0.0004). The same was the case for the patients with vestibular symptoms at LFU (Figure 3).

The deterioration in vestibular symptoms showed a positive correlation with the gradual deterioration in the DHI (chi-square *p* = 0.028). However, there was no correlation between the gradual improvement in DHI and the improvement in vestibular symptoms (chi-square *p* = 0.17).

## 4. Discussion

Several studies [7,14,15] have shown that dizziness and vertigo affect the quality of life (QOL) of VS patients more than hearing loss or facial neuropathy. Hearing loss in VS patients can reliably be measured using well-established methods such as pure-tone audiometry, speech audiometry, or otoacoustic emissions [16]. Conversely, the quantification and objectification of vestibular symptoms represents a diagnostic challenge and requires a multidimensional approach, as the underlying etiology is often difficult to assess and may comprise neurological, psychological, and cardiovascular comorbidities of varying intensity. Although several test protocols for instrument-based diagnostics such as the caloric test or electronystagmography are available, these tests are inconsistently and infrequently applied.

Vestibular symptoms are difficult to assess and interpret by clinical examination alone, and VS patients reporting about “dizziness” often cannot separate objectively between vertigo, postural imbalance, or psychological aspects of their imbalance [17]. In addition, the perceived impairment of VS patients caused by vestibular symptoms is difficult to assess and to compare among patients. The PANQOL is a reliable scale and valid disease-specific quality-of-life instrument for vestibular schwannoma. However, as this questionnaire covers the entire symptom complex of vestibular schwannomas and is not specific to vestibular symptoms, it was not used in our study [18]. Regarding vestibular symptoms, there are only a few standardized reporting tools available, which encompass the Vertigo Symptom Scale (VSS), Visual Vertigo Analogue Scale (VVAS), or Dizziness Handicap Inventory (DHI). The latter is the most commonly used instrument and has been validated for consistency and reliability, and has been routinely applied in patients with peripheral vestibular disorders. With regard to VS patients treated with SRS, the DHI has so far only been used in a few series of patients undergoing Gamma Knife SRS [10,19,20]. In addition, published studies investigating dizziness and vertigo in VS patients vary widely regarding the length of follow-up and mostly lack long-term data [21]. For this reason, we here for the first time applied the DHI as a longitudinal and standardized reporting tool before and after robotic SRS of unilateral VS. All patients completed the DHI correctly so that each questionnaire could be evaluated. This shows the usability of this instrument and is in line with former research [22].

We found an improvement in the total DHI score in about 30% of our cohort during follow-up, which compares well with a study by Wackym et al. [20], who examined 55 patients with sporadic VS. The majority of patients did not suffer from significant vertigo either before or after Gamma Knife SRS. Only the group of elderly patients (>65 years of age) showed a significant improvement in the total DHI score. In contrast to these results, we did not observe an improvement in DHI influenced by age (Table 3). In addition, we found that the DHI total score changed significantly over time. A significant factor for the improvement in the DHI score after SRS was the duration of the FU period, as also shown by Wackym et al. and Pollock et al. [19,20]. The authors conclude that long-term follow-up is necessary to predict the course of vestibular dysfunction in VS patients and to detect improvements [19,20].

Neither deterioration nor improvement in DHI total score was influenced by any of the tested variables (Table 3). At least with regard to the dose, this finding is not necessarily surprising, since the dose parameters of the tumor itself were tested here. To what extent the radiation exposure of the vestibule plays a role remains still unclear. To date, several studies [23] have examined hearing preservation as a primary endpoint with regard to the radiation dose administered to the cochlea. It has been shown that a low cochlear dose is beneficial for the long-term preservation of hearing [23,24]. In contrast, only one study to date [25] has investigated the influence of vestibular radiation dose on vestibular outcome measured by vestibular tests and subjective dizziness. The authors report that a vestibular dose D_min_ of 5 Gy and above worsened dizziness in patients. In contrast, the D_mean_ and D_max_ received by the vestibule were significantly lower in patients who had improved caloric function. These results suggest that the radiation dose may have an effect on vestibular symptoms after SRS. Unfortunately, the study did not use a standardized questionnaire such as the DHI, which makes it difficult to compare subjective symptoms.

### Pros and Cons of the DHI Score and Classification

Studies have shown that the results of the subscales in the DHI questionnaire to determine the degree of dizziness are biased in certain cases. One example is that a patient’s avoidance behavior may suggest improvement [26]. In addition, Cohen et al. [27] points out that the use of three response options may limit the ability to register small changes and may not obtain the full range of disability experienced by patients. Regarding the measurement properties of the DHI, a systematic review by Koppelaar-van Eijsden et al. [28] shows suboptimal content validity and internal consistency. The authors conclude that the DHI should never be used alone, but only in conjunction with other outcome parameters. Therefore, we here correlated the DHI results with the unstructured patient self-reported outcomes assessed during routine clinical follow-up. Nevertheless, several studies that use the DHI as an outcome assessment for vestibular symptoms found the DHI responsive to changes during FU [29,30,31]. For instance, the study of Zanardini et al. [31] used the DHI to assess vestibular rehabilitation and showed a significant improvement in correlation with clinical status. In most cases of cited studies [29,30,31], however, the mean change in score has fallen below the minimum change of 18 points. In our study, this leads to the confusing fact that there are patients who improve from the “moderate” to “mild” category with only a two-point change in DHI score. In the original work of Jacobson and Newman, the authors recommended a change of 18 points to be 95% certain of a true change [12]. We implemented this recommendation in our study accordingly, considering only a difference of at least 18 points in total DHI scores as a true change. It is therefore possible that patients improving from one category to another (e.g., “moderate” to “mild”) are not necessarily considered having experienced a true change in their vestibular symptoms. Thus, the four DHI categories (“none” to “severe”) are not particularly precise for mapping a real change and cannot be understood solely as outcome parameters.

A study of Duong et al. [22] validated the German version of the DHI, which was used in our study in a longitudinal evaluation of 50 patients. The patients included in the previous study showed a high level of compliance in answering the questionnaire. This was also the case in our study. Finally, the authors concluded that the DHI is a viable option for the assessment and monitoring of treatment outcomes. Further, the authors found the DHI to be useful for documentation of patients’ disorders, and thus for quality assurance. The latter is especially important for SRS against the background of the associated dose exposure of organs at risk like the vestibule.

## 5. Conclusions

In summary, we were able to show that the total DHI scores in patients treated with SRS significantly improved during follow-ups. However, only a minority of VS patients suffered from severe DHI scores before and after SRS. The improvement in vestibular symptoms as documented by the application of a valid questionnaire in VS patients treated with SRS could enable clinicians to better understand and anticipate the frequency, severity, and development of vestibular symptoms in a consistent manner. In addition, our study has proven that the DHI is a feasible instrument in collecting these data on vestibular symptoms in a standardized and comparable way. To further refine data of the impact of RS dosimetry on vestibular function, studies using standardized reporting tools like the DHI are particularly important, leading to more homogeneity and comparability of the study results.

## Figures and Tables

**Table 1:** Clinical characteristics and treatment parameters of patients. Data are presented as median values with the range in brackets.

**Patient Characteristics**	
Total no. of patients	128
Gender (m:f)	54:74
Recurrent AN	17 (13%)
No. of Koos-Grade	Koos I: 30 Koos II: 49 Koos III: 43 Koos IV: 5
Clinical and radiological FU (months)	36 (12–106)
Age (years)	60 (range: 20–82)
Tumor volume (cm^3^)	0.95 (range: 0.04–7.1)
median FU (months)	36 (range: 12–106)
**Initial Symptoms and Signs**	
Acute hearing loss (%)	25 (19%)
Hearing disturbance (%)	101 (79%)
Vestibular symptoms (Vsym) (%)	81 (63%)
Deafness (%)	14 (11%)
CN V impairment (%)	5 (4%)
CN VII impairment (%)	14 (11%)
**DHI Categories before Treatment**	
None (0–14)	48 (38.3%)
Mild (16–34)	36 (28.1%)
Moderate (36–52)	17 (13.3%)
Severe (54–100)	26 (20.3%)
**Radiation Parameters**	
Marginal dose (Gy)	13 (range: 12–14)
Dose prescription, isodose (%)	80 (range: 65–82)
Coverage (%)	99.6 (range: 94–100)
D_min_	12.6 (range: 9.3–13.2)
D_mean_	14.7 (range: 12.7–16.8)
D_max_	16.4 (range: 15–20)
nCI	1.19 (range: 1.04–1.6)

**Table 2:** Statistical results of a binary logistic regression showing that the true change (≥18 points) of the DHI score in the direction of improvement or deterioration is not influenced by any of the tested variables (*p* > 0.05).

	*p*-Values (Binaric Logistic Regression)	
Variable	DHI Improved (n = 22/73)	DHI Declined (n = 11/120)
Age	0.550	0.920
Gender	0.867	0.861
Pre-treatment	0.474	0.200
Tumorvolume	0.408	0.998
Dmax	0.252	0.762
Dmean	0.754	0.881
Dmin	0.561	0.138
Coverage	0.634	0.255
nCI	0.128	0.620

**Table 3:** Chi-square test shows a correlation of the categories DHI 0–1 (none–mild) and DHI 2–3 (moderate–severe) with the incidence of Vsym before SRS (*** *p* = 0.002) and at LFU (** *p* = 0.008).

**Initial Vsym**	**DHI 0–1**	**DHI 2–3**	**Total**
yes	46	35	83 ***
no	39	8	45 ***
**Vsym at LFU**	**DHI 0–1**	**DHI 2–3**	**Total**
yes	51	29	80 **
no	41	7	48 **

## Data Availability

The data presented in this study are available on request from the corresponding author. Due to the data protection of the patients examined in this study, the data is only made available in anonymised form.
